# Supplementary material for: Deriving time-concordant event cascades from gene expression data: A case study for Drug-Induced Liver Injury (DILI)
Source: PLoS Comput Biol. 2022 Jun 10;18(6):e1010148. doi: 10.1371/journal.pcbi.1010148 (PMC9292124; doi:10.1371/journal.pcbi.1010148)
Supplement: S5 Table — For TF events which are significantly enriched before or at adverse histopathology, known interactions supported by time concordance are shown. With respect to the interaction, the absolute and relative frequency are shown for how often the source TF was observed “before” or “before or at” downstream TF activity. Additionally, the source of the interactions provided in Omnipath are shown for protein-protein interactions and the DoRothEA confidence level for TF-target gene interactions. (DOCX) [file pcbi.1010148.s005.docx]

| class | Preceding event | Later event | TPR (Before or at) | TPR (Before) | Sources |
| --- | --- | --- | --- | --- | --- |
| PPI | Mef2c(Down) | Myod1(Down) | 0.667 (4/6) | 0.333 (2/6) | BioGRID;Lit-BM-17;SIGNOR;Wang |
|  | Nr1h2(Down) | Ppara(Down) | 0.3 (3/10) | 0.1 (1/10) | SignaLink3 |
|  | Pax6(Down) | Maf(Down) | 0.333 (3/9) | 0.111 (1/9) | SPIKE |
|  | Ppara(Down) | Nr1h2(Down) | 0.444 (4/9) | 0.222 (2/9) | SignaLink3 |
| Regulon | Cebpa(Down) | Hnf4a(Down) | 0.333 (3/9) | 0 (0/9) | A |
|  | Elf3(Down) | Meis1(Down) | 0.364 (4/11) | 0 (0/11) | C |
|  | Hnf1a(Down) | Hnf4a(Down) | 0.667 (6/9) | 0 (0/9) | A |
|  | Hnf4a(Down) | Cebpa(Down) | 0.75 (3/4) | 0 (0/4) | A |
|  | Nfe2l1(Down) | Tead1(Down) | 0.727 (8/11) | 0 (0/11) | C |
|  | Nr1h2(Down) | Srebf1(Down) | 0.222 (4/18) | 0 (0/18) | C |
|  | Nr1h3(Down) | Srebf1(Down) | 0.5 (9/18) | 0.278 (5/18) | C |
|  | Pbx2(Down) | Meis1(Down) | 0.636 (7/11) | 0 (0/11) | C |
|  | Pbx3(Down) | Meis2(Down) | 0.636 (7/11) | 0 (0/11) | C |
|  | Pdx1(Down) | Hnf4a(Down) | 0.667 (6/9) | 0.444 (4/9) | C |
|  | Prdm1(Down) | Tead1(Down) | 0.364 (4/11) | 0 (0/11) | C |
|  | Rara(Down) | Hnf4a(Down) | 0.222 (2/9) | 0 (0/9) | A |
|  | Rela(Up) | Nfkb1(Up) | 1 (4/4) | 0 (0/4) | A |
|  | Sox11(Down) | Tead1(Down) | 0.273 (3/11) | 0 (0/11) | C |
|  | Tal1(Down) | Nfkb1(Up) | 0.25 (1/4) | 0 (0/4) | A |
|  | Tcf12(Down) | Tead1(Down) | 0.818 (9/11) | 0 (0/11) | C |
|  | Tcf4(Down) | Tead1(Down) | 0.545 (6/11) | 0 (0/11) | C |
|  | Zfp384(Down) | Meis2(Down) | 0.636 (7/11) | 0 (0/11) | C |
|  | Zfx(Up) | Zfx(Up) | 1 (10/10) | 0 (0/10) | E |
