# Supplementary figures and images for: Deriving time-concordant event cascades from gene expression data: A case study for Drug-Induced Liver Injury (DILI)
Source: PLoS Comput Biol. 2022 Jun 10;18(6):e1010148. doi: 10.1371/journal.pcbi.1010148 (PMC9292124; doi:10.1371/journal.pcbi.1010148)

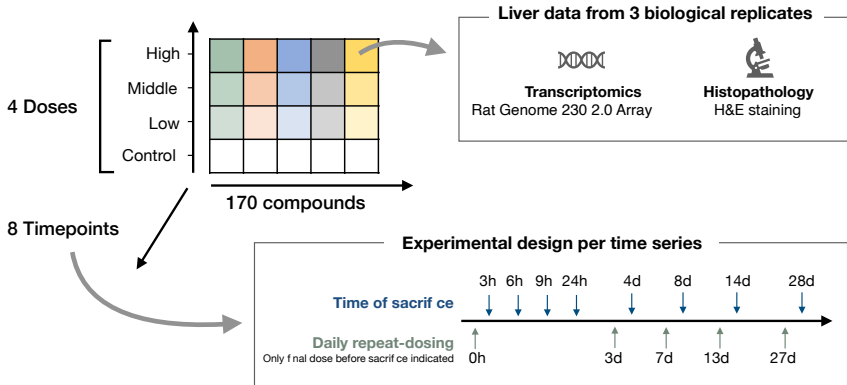

Supplement: S1 Fig — 6-week-old male Crl:CD Sprague-Dawley (SD) rats were treated with a range of compounds using daily repeat-dosing. For each compound, four doses were used including a vehicle control, and samples were taken at 8 timepoints. For each combination of compound, timepoint and dose, histopathology was annotated and gene expression measured for 3 replicates. (PDF) [file pcbi.1010148.s006.pdf]

Number of experiments

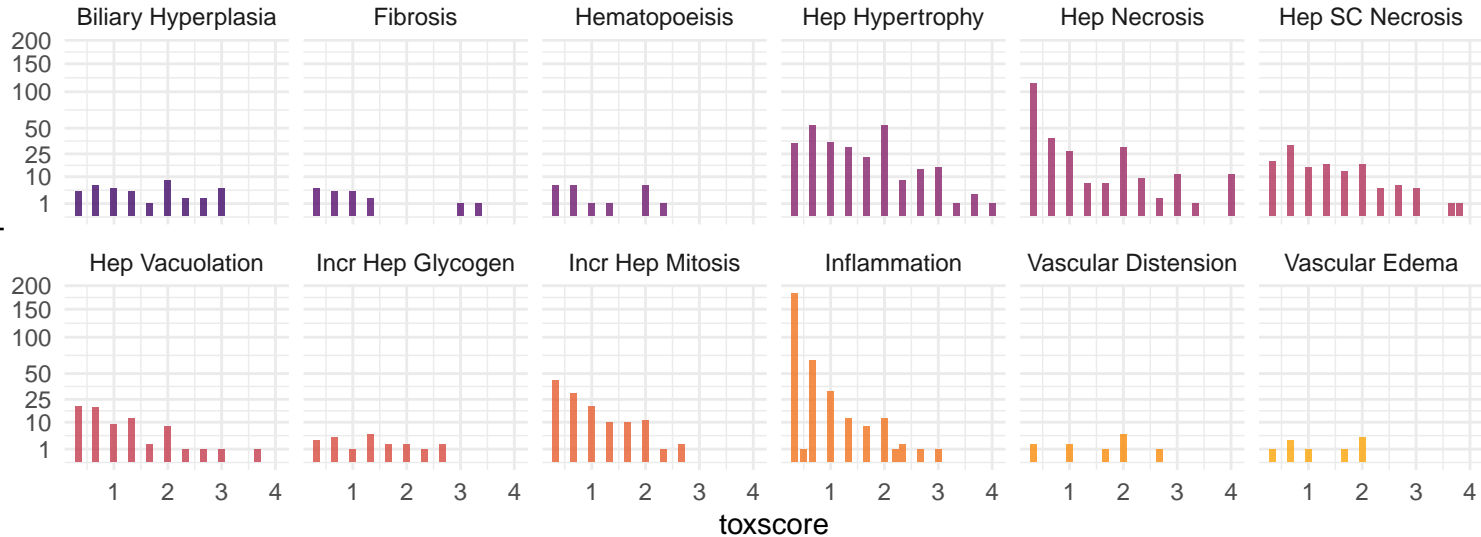

Supplement: S2 Fig — (PDF) [file pcbi.1010148.s007.pdf]

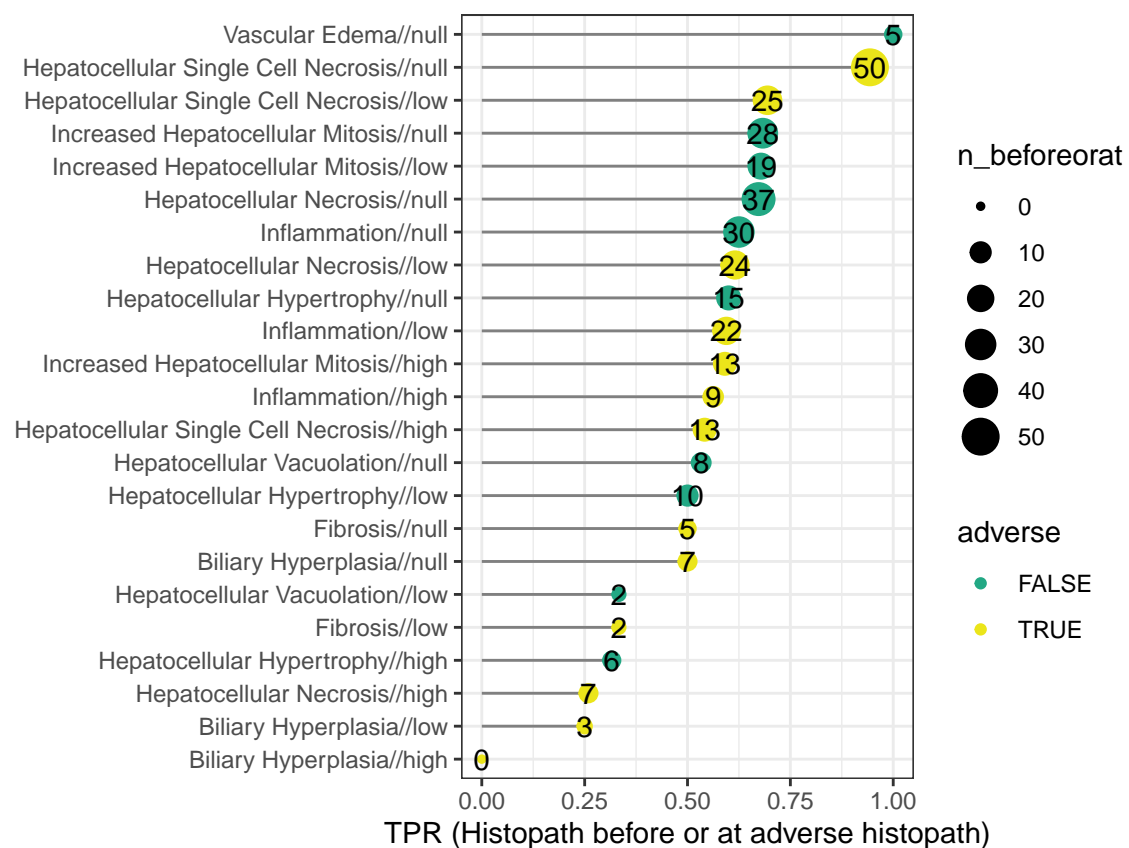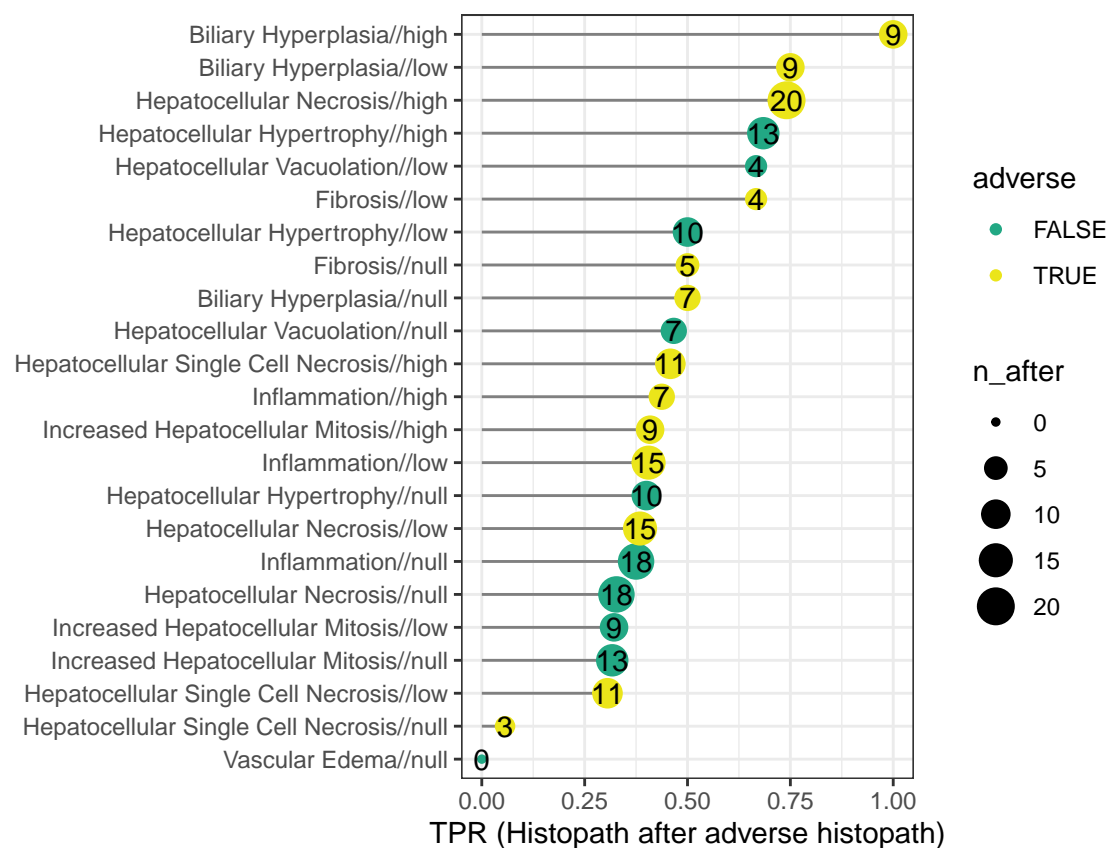

Supplement: S3 Fig — For adverse and non-adverse histopathological findings, the frequency before or at first adverse histopathology is shown (left). For adverse findings, this indicates how frequently they were one of the first adverse histopathological findings given that they cannot occur before by definition. This identifies single-cell necrosis at any severity (“null”), as the most frequent finding, both in absolute and relative terms. (PDF) [file pcbi.1010148.s008.pdf]

# Pathways

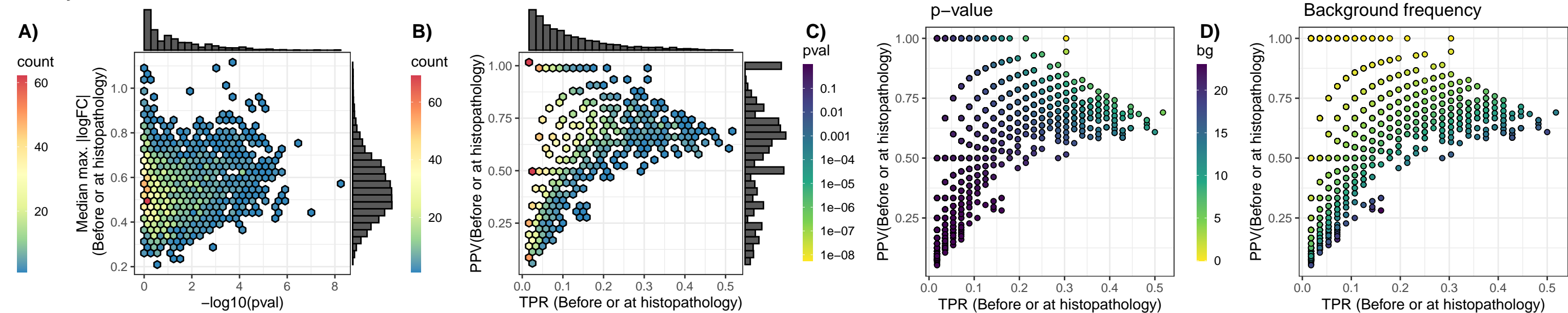

# Transcription Factors

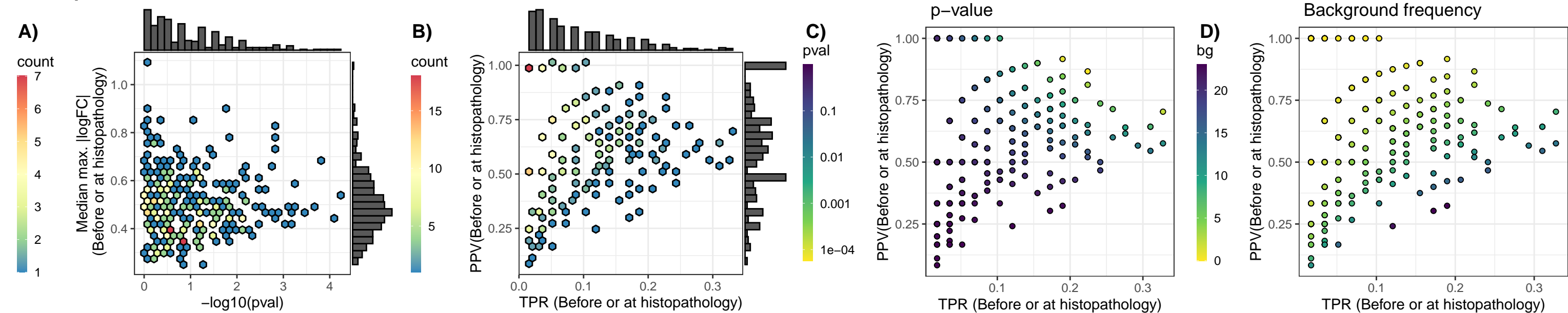

Supplement: S4 Fig — The dependency between different metrics is shown. A) Frequency of events by median max. |logFC| before or at histopathology and enrichment p-value. B) Frequency of events by true positive rate (TPR) and positive predictive value (PPV) before or at adverse histopathology. C) Direct relation between TPR, PPV and enrichment p-value. D) Direct relation between TPR, PPV and frequency in background time-series. (PDF) [file pcbi.1010148.s009.pdf]
