# Supplementary material for: Deriving time-concordant event cascades from gene expression data: A case study for Drug-Induced Liver Injury (DILI)
Source: PLoS Comput Biol. 2022 Jun 10;18(6):e1010148. doi: 10.1371/journal.pcbi.1010148 (PMC9292124; doi:10.1371/journal.pcbi.1010148)
Supplement: Attachment — Submitted filename: point2point_AB.docx [file pcbi.1010148.s012.docx]

Dear Dr. Gallo and Dr. Alber,

We herewith submit the manuscript entitled “Deriving time-concordant event cascades from gene expression data: A case study for Drug-Induced Liver Injury (DILI)” by Liu et al. for publication as research article at *PLOS Computational Biology*.

We’d like to thank all reviewers for their generally positive and constructive comments and have revised the manuscript based on the these as outlined below. We are confident that the approaches discussed in our research article would appeal to the readership of *PLOS Computational Biology*.

Thank you very much for your consideration.

With best regards,

Anika Liu

## Reviewer #1

Reviewer #1: This manuscript introduces “first activation” as the method to quantify time concordance to provide evidence for causality. The authors perform a case study on a liver dataset from repeat-dose study in rats using the gene expression and histopathology data. This manuscript uses known evidence and prior biological knowledge in DILI to validate the proposed methodology and points out some potential new TF interactions.

I think this manuscript is well-written and organized but could be improved with some analysis aided. I have following detailed comments.

We’d like to thank the reviewer for their generally positive comments.

General comments:

1. The key concept of this manuscript is “first activation” and the authors argue that this is generalizable and automatable to other topics. However, the discussion on this concept is quite limited, and should be extended. How the authors defined “first activation” was only mentioned in Methods and should be written in the main text. The threshold seems to be very important as this could lead to distinct results and authors should discuss how robust the current threshold is.

Thank you for these comments, which we fully acknowledge. There are indeed multiple thresholds involved in this study which we needed to make clearer to the reader. Firstly, the histopathology scale is defined by severity thresholds. Secondly, significance thresholds are used to evaluate activation of gene-expression based events. While we regard the third cut-off, an FDR of 0.05 for the activation of gene expression-based events, as commonly established, the other two thresholds are indeed specific to this study although we aimed to choose this in an objective way.

We now provide additional information on why the given histopathology thresholds were chosen (page 9, l. 214 - 219):

“We then evaluated which histology groups were frequently found in the adverse compound-dose combinations (observed in >10% of adverse time series, corresponding to at least 5 out of 40 cases) with at least 50% of findings being in adverse time series (Fig 3A). All of the included histology groups are significantly enriched in adverse conditions, however, these criteria were implemented to identify findings with a certain specificity and frequency instead of allowing a trade-off between both.”

We discuss that this classification is indeed difficult to justify and that there is in fact generally discrepancy between annotations, even between the 2 classifications by the FDA based on post-marketing data (DILIst and DILIrank). Being aware of this, but requiring a definition to perform analysis we point to our Shiny app where alternative definitions, including alternative thresholds, can be explored:

“We are aware that also broader or more targeted phenotypes might be of interest, and we hence provide a Shiny app where results for alternative definitions of adverse and non-adverse histopathology groups can be explored.”

2. The authors mainly used 10 known events in DILI for their case study, but as they mentioned, they identified hundreds of both pathway-level and TF-level events in the datasets. Although using well-known events to validate the methodology is very much needed, analysis on some other less well-studied events as examples should be also discussed.

We have extended our discussion of events derived in a data-driven way in “Data-driven prioritization of cellular events taking place before adverse histopathology” which focusses on the top 10 pathways and TFs by logFC, significance and frequency, respectively. We would also like to highlight that the subsequent TF and time dependence analysis are not limited to the 10 events but instead explore all pathways and TFs with evidence for preceding adverse histopathology.

Minor points:

1. This manuscript focuses on one case study, a liver dataset from repeat-dose studies in rats, a figure showing the concept of this experiment, depicting the info about collecting timepoints, doses etc is helpful and needed.

Added as S1 Fig and extended Methods.

2. To establish causality, there are multiple rules in Bradford-Hill criteria, while this manuscript focuses only on temporal order, the other rules should be discussed to infer causality.

This was previously only discussed in the introduction, but is now also included in “limitations of the study” (page 28, l.761 - 768):

“Lastly, it should be highlighted that time concordance is necessary for casual relations but not sufficient to prove it. For instance, two events may be time-concordant because they are causally linked to a shared preceding cause. To distinguish these effects, the additional Bradford-Hill considerations can be helpful, but only prior knowledge has been considered in parts of this study. In particular essentiality would provide strong evidence for causality, however, requires targeted experiments and hence is unsuitable for hypothesis generation. In contrast, dose and incidence concordance are generally feasible from a data-driven standpoint but were not pursued in this case study due to the low number of doses and replicates. “

3. Results that used method described in Method section should be indicated.

We have addressed this point in the revision by introducing the workflow including figure (Fig 2) at the beginning of results and also referring to the Methods section (p.7, l.179-181):

“In order to derive the time concordance between cellular events and later adverse histopathology, we use the workflow outlined in Figure 2 with each step being also introduced in the subsequent sections and details on their respective implementation in Methods.”

We also refer to Methods when pathway/TF activation as well as time concordance are first introduced (p.11, l.292):

“To identify cellular mechanisms in the early pathogenesis of DILI, we next studied time-concordant cellular changes preceding later adverse histopathology (see Methods).”

4. Line 72, AE was first mentioned and need full descriptions.

🡪 Added

5. Fig 1, defining A,B,C,D as candidates preceding events in panel A) causes confusion in panel B), where B is defined as the potential later event.

Renamed events in panel B.

6. More descriptions needed for S1 Table, not clear what + or ++ means.

The +/++/etc. have been replaced with descriptions of the strengths and weaknesses of the models.

7. Reasoning behind choosing 10% and 50% as the threshold needed.

This was previously only included in Methods (p.31, l.851 ff):
“We defined findings as adverse histopathology if they are observed in at least 5 out of 40 adverse time-series to remove rare histopathological findings, and additionally require that at least 50% of findings are in adverse conditions to remove findings which are unspecific. All labels which were identified with these criteria are significantly enriched among time-series labelled as adverse by Sutherland et al. in comparison to those that were considered non-adverse using a one-sided Fisher’s Exact test (p-value > 0.0001), performed using the fisher.test function of the stats R package. However, this combination of additional criteria was chosen to exclude findings which are rare or weakly associated.”

This reasoning has now added it into the main text as well (page 9, l.217-219):
“All of the included histology groups are significantly enriched in adverse conditions, however, these criteria were implemented to identify findings with a certain specificity and frequency instead of allowing a trade-off between both.”

We discuss that this classification is indeed difficult to justify and that there is in fact generally discrepancy between annotations, even between the 2 classifications by the FDA based on post-marketing data (DILIst and DILIrank). Being aware of this, but requiring a definition to perform analysis we point to our Shiny app where alternative definitions, including alternative thresholds, can be explored (p.9, l.226ff):

“While we focus on the described definition of adverse histopathological findings in this study, the difficulty in summarising a complex phenotype such as DILI into a binary classification, adverse or not adverse, is well established [27,28] and is also demonstrated by the discrepancies between DILI classifications from DILIst, DILIrank and those derived by Sutherland et al based on the TG-GATEs data (S1). We are aware that also broader or more targeted phenotypes might be of interest, and we hence provide a Shiny app where results for alternative definitions of adverse and non-adverse histopathology groups can be explored.”

8. Fig2A, unclear about what is Number of adverse conditions vs Fraction of adverse conditions.

Rephrased caption: “Histopathology labels are defined for each histopathological findings at 3 different toxscore cut-offs, namely “null” (toxscore>0), “low” (toxscore>0.67) and “high” (toxscore>1.34. For each label, the number of occurrences in the 40 adverse time series and the fraction of adverse time series among all occurrences of the given histopathology label are shown.”

9. Fig3 right panel, texts overlap.

The figure was regenerated and texts aren’t overlapping anymore.

10. Fig 4, the figure should be reorganized. The text of ‘Up-regulated’ and ‘Down-regulated’ are unnecessary since there are color codes for it. The order of these ten events is reversed as Fig 3. Why oxidative stress is missing?

Oxidative stress is missing because none of the gene sets were significantly time-concordant, and we perform downstream analysis only for those.

We clarified this in the text by first adding a sentence that oxidative stress was not significant (p.13, l.352-254):

“In contrast, the opposite was found for oxidative stress with the Jun Proto-Oncogene (Jun) being one of the most significantly enriched gene-level events but lacking significant changes on the gene-set level“

We also clarified that only time-concordant events are considered (p. 13, l.363-366):

“Firstly, we investigated how strongly pathways were dysregulated comparing the maximal absolute log fold changes (|logFCs|) before or at adverse histopathology in each adverse time-series for significantly time-concordant events (Fig 5).”

The order of events has been changed in Fig.3.

12. Fig5, ‘If the event was over- represented before adverse histopathology (p-value < 0.05) the point was additionally circled in black’, very hard to see the black circle in figure, need to change to a different coloring scheme.

In fact all shown events were significant, so all were circled and the sentence itself was unnecessary to include. It has been removed now.

13. Fig 5, there are a lot of individual genes are highlighted, but the text did not mention why they were highlighted.

A paragraph was added about this (page 15, l.403 ff):

“Due to the previously discussed complementarity of gene- and gene set-level analysis, we also show TPR and PPV for individual genes with a focus on those which are involved in gene sets mapping to known key events. The most significant genes, already highlighted in Fig 4, reveal a high frequency for the up-regulation of the Acyl-CoA thioesterases Acot2, Acot3 and Acot4, as well as the for the Enoyl-CoA Hydratase 1 Ech1 which aligns with the relatively high frequency of pathway-level events linked to mitochondrial and peroxosomal processes. Furthermore, the most frequent gene-level events with a PPV=1 are the up-regulation of Activating Transcription Factor 3 (Atf3) which was found to promotes hepatic fibrosis [38] and Enoyl-CoA Delta Isomerase 1 (Eci1).”

14. The captions and legends of figures in this manuscript need substantial improvement. The audience should be able to get the key ideas from the figure by looking at the figure and caption and legends.

Figures and caption have been improved as described in the replies to the other comments in detail.

15. Fig 8B, description on the legend should be clearer in the captions. For example, ‘active_tf’ should be described. Also the axis label ‘TPR(Expr|Before or at TF&adverse)’ needs description.

We updated the caption as follows (p.21):

“Fig 9: Transcription Factor (TF) activity and expression before adverse histopathology. A) Significance of enrichment in adverse conditions for matched TF activity and expression-based events. Events only found on the expression or TF level are not included in the figure due to the inability to perform a statistical test for those. B) For significantly enriched TF activity-based events, the True Positive Rate (TPR) of observing TF activity before or at the time of adverse histopathology is shown, as well as the TPR for observing TF expression changes before TF activity in the time series where it precedes adverse histopathology. As additional information the absolute number of time series in which TF activity is found before or at adverse histopathology is indicated as circle size.”

16. The upper or lower case of term (e.g. TF) should be kept consistent between figures and texts. For example, Line 373, ATF4 while in Fig 8B it’s Atf 8. There are many other places having this inconsistency.

This inconsistencies stem from the fact that different gene symbols are used for rat and human genes with the same gene in different species being sometimes meant in one sentence. We now when we are referring to homologous genes in different species (p.22, l 593 ff):
“The event with the 2nd highest relative frequency of expression preceding TF activity as well as the highest frequency of TF activity preceding injury is Atf4, for which expression of the homologous gene in humans is known to be induced as part of the ER stress response contributing to adverse liver phenotypes”

17. Line 375, any idea why Ahr never preceeds TF?

We have added a biological explanation on this.(p.22, l.596):

“In contrast, it was found that for the Aryl Hydrogen Receptor (Ahr) and the Peroxisome Proliferator-activated Receptor Alpha (Ppara) changes in expression never preceded those in TF activity which aligns with their roles as nuclear receptors which are generally post-translationally activated via ligand binding [58,59].” (p.22)

(Ppara was identified after fixing the gene-level statistics as outlined in XXXX)

18. Fig 9. Lighter gray and darker gray are very hard to distinguish, choose another color legend.

The figure has been revised according to the referee’s comment, see new Fig 10 (previously Fig 9).

## Reviewer #2

Reviewer #2: Reviewer’s Report

Title: Deriving time-concordant event cascades from gene expression data: A case study for Drug-Induced Liver Injury (DILI)

Reviewer's report:

Authors of this manuscript study time concordance pathway cascades using the concept of first activation to generate hypotheses on potentially causal mechanisms following Bradford-Hill criterias. They use liver data from repeat-dose studies in rats to study time concordant gene expression-derived events preceding adverse histopathology, which serves as surrogate readout for Drug-Induced Liver Injury (DILI). While this manuscript is based on an interesting hypothesis, the manuscript could be improved.

We’d like to thank the reviewer for their generally positive comments. Detailed points have been addressed as described in the following.

Major Compulsory Revisions:

This study uses association analysis to discover the mechanisms of adverse outcome pathways. When a cause–effect relationship can be established based on first activation,

How do authors discriminate direct effects from indirect effects, especially with limited time points with gaps in time? For example, gene C is an unobserved confounder for the association between gene A and B but A is not a cause of B.

We agree that the limited time points indeed mean that concordance is difficult to evaluate within single time-series, which is why sets of time-series are considered (p.5, l. 132 ff):

“Although the confidence of these temporal orders per time series is limited by the noisiness of gene expression data and the low time resolution, statistical significance can be evaluated across time series and we only consider relations time-concordant if the preceding event is significantly more frequently found before the later event than in time series without (Fig 1B).”

It is true that direct and indirect effects may not be distinguishable and discuss this in the limitations of the study (page 28, l.761 - 768):

“Lastly, it should be highlighted that time concordance is necessary for casual relations but not sufficient to prove it. For instance, two events may be time-concordant because they are causally linked to a shared preceding cause. To distinguish these effects, the additional Bradford-Hill considerations can be helpful, but only prior knowledge has been considered in parts of this study. In particular essentiality would provide strong evidence for causality, however, requires targeted experiments and hence is unsuitable for hypothesis generation. In contrast, dose and incidence concordance are generally feasible from a data-driven standpoint but were not pursued in this case study due to the low number of doses and replicates.“

It is true that everything that is time-concordant is causal, but everything that is causal is time-concordant. Therefore, the presented approach provides some evidence towards causality which is not generally true for previous work on cpAOPs (p.4, l.87 ff) which integrates statistical associations form different contexts and neither for previous work in toxicogenomics (p.5, l.119 ff):

“Previous studies focussed on the time (and dose) dependence of gene expression-derived events in the context of adverse findings [21,22], so the changes of individual events across changes in time (and dose), and also aimed to predict later adverse findings from fixed early timepoints [14,15]. From a mechanistic perspective, however, neither activation at a certain timepoint nor a certain progression over time is mandatory, but only time concordance, so activation of the key event before the downstream adverse effects.”

How do authors account for transient changes of the genes due to stochastic variations or biological fluctuations from unwanted external factors that may be random?

We only consider events as time-concordant if changes are more frequent than expected as outlined in Methods (Temporal concordance of events), which means that we are accounting for the fact that event activation might take place independently. This fact is discussed in the introduction and was extended now to make this point more clear (p.5, l.132 ff):

“Although the confidence of these temporal orders per time series is limited by the noisiness of gene expression data and the low time resolution, statistical significance can be evaluated across time series and we only consider relations time-concordant if the preceding event is significantly more frequently found before the later event than in time series without (Fig 1B). Furthermore, it is possible to separate out events which depict general perturbation response but are unspecific, as well as rare events, which are predictive but only observed for a small subset of compounds.”

The influence by unwanted external factors should be limited given that all experiments were performed using the same protocols. We believe that bias may be present due to the compounds selected which naturally only depict certain modes of toxicity. This is also discussed in the limitations section (p. 27, l.731 ff):

“This does not only mean that rare events might be missed as they occur between measured timepoints and that small effects might not be identified as significant, but also that there is potentially a bias based on the tested compounds towards the represented modes of toxicity.”

Some genes may appear too significant because of their role as network hub genes in the dataset that are involved in many biological processes. These hub genes are connected to many upstream regulators so their occurrence in cause-effect relationship could be general and unspecific unless integrated with prior knowledge. I would like to hear the author's response on how this could affect their inferences.

Given the setup of the time concordance analysis, which focusses on TFs and pathways and is estimated by gene set activity, this should not be affected by network hub genes. It is true that certain events are generally important and more likely dysregulated than others (for biological or maybe also other reasons), however, we evaluate the enrichment in adverse time series in comparison to background time series exactly due to this reason, namely to account for general and unspecific responses. What is truly biased is the prior knowledge network which we use to infer TF-TF interactions (Fig.10). Here, Tead1 was identified to have multiple links to upstream TF with low confidence, which may be biased by the fact that it is well connected. So in this context, it is in fact the integration with prior knowledge which might treat hub nodes (in the prior knowledge network) preferentially. However, the effect is limited by the integration with time concordance which is data- and not knowledge-driven and prior knowledge on causal interactions should be leveraged despite potential bias.

I) Methods:

Some of the important details of the methods are scattered in results and method sections. The methods section of this manuscript could be further improved by including details on methodology. Please refer to my comments below. I would strongly recommend a flowchart to improve readability.

We have now added a flowchart as Fig.2 and also provided more details on the methodology, based on the following suggestions by the reviewer.

Authors state in the abstract section: “from the TG-GATEs database which comprises measurements across eight timepoints, ranging from 3 hours to 4 weeks post-treatment”. How frequent are the time points? How does the gap in time points affect false predictions? Also, it is not clear how many biological replicates are used for different time points*dose.

We have added information on the TG-GATEs study design in S1 Fig now, including the number of replicates and timepoints measured. The timepoint coverage is further described in the Methods section (p.30, l.817 ff):
“Only the 360 compound-dose combinations with at least 6 measured timepoints after quality control were included. Out of the these all eight timepoints were measured in most time series, while only six timepoints were measured in two time series, and only seven timepoints in seven time series. “

It is true that events which take place between measurements can be missed. We discussed this as one of the limitations of the study (p. 27, l.730):
“However, the present analysis is based on a limited number time-series as well as only few timepoints within each time-series. This does not only mean that rare events might be missed as they occur between measured timepoints and that small effects might not be identified as significant, but also that there is potentially a bias based on the tested compounds towards the represented modes of toxicity. “

While abstract mention TG-GATEs database which comprises measurements across eight timepoints, ranging from 3 hours to 4 weeks post-treatment, the results section states: “These adverse histopathology labels were next used to define 61 time-series associated, covering 38 compounds, as adverse (S2 164 Table).” It is not clear whether authors used 61 time series data which had similar time points.

We now explain this better in the results section and fixed an error in the text (p. 9, l. 235 ff):

“Next, out of all 360 time-series with at least 6 measured timepoints, the 61 time-series in which any of the adverse histopathology labels is found were identified, which covered 38 compounds (Table S2).”

More info about the timepoints per time series is provided in the methods section (p.30, l.817 ff):

“Only the 360 compound-dose combinations with at least 6 measured timepoints after quality control were included. Out of the these all eight timepoints were measured in most time series, while only six timepoints were measured in two time series, and only seven timepoints in seven time series.”

“To evaluate which pathway or TF is dysregulated, we computed the differential activity in comparison to the time-, vehicle-, and experiment-matched control group using limma and identify significantly dysregulated gene sets with a False Discovery Rate (FDR) < 0.05.” What are the exact comparisons authors performed?

This has been rephrased (p.32, l.884): “To evaluate which pathway or TF is dysregulated, we computed the differential activity in comparison to the vehicle control group, which was treated for the same amount of time and as part of the same experiment, using the moderated t-statistic in limma [83].”

“For pathways and TFs, we defined first activation as the earliest time of measurement at which significant differential regulation was observed (FDR < 0.05) in each direction, while an additional logFC cut-off has been implemented for individual genes” For pathways and TFs, we defined first activation as the earliest time of measurement at which significant differential regulation was observed (FDR < 0.05) in each direction, while an additional logFC cut-off has been implemented for individual genes.” It is not clear how first activation of a pathway is defined? Do you consider a pathway to be activated even if one TF is expressed?

A pathway is considered first activated at the earliest when there is differential activity of the gene set as inferred with GSVA. This is explained in the preceding section (Pathway and TF activity inference, p.32, l.873 ff) and has been extended to make it clearer. We additionally added a workflow figure (Fig. 2) in order to make the overall process more easily understandable to the reader.

II) Results:

“These adverse histopathology labels were next used to define 61 time-series associated, covering 38 compounds, as adverse (S2 164 Table)”: What were the initial time-series and how did you define 61 time-series based on histopathology labels. If authors removed time points for downstream analysis, how does it bias the predictions?

The time-series with adverse histopathology were defined as follows (page 9, l.235 ff):

“Next, out of all 360 time-series with at least 6 measured timepoints, the 61 time-series in which any of the adverse histopathology labels is found were identified, which covered 38 compounds (S2 Table).”

Timepoints for downstream analysis were not removed, but in some cases a lower number of timepoints were available, we have clarified this in the methods section (p. 30, l.817 ff):

“Only the 360 compound-dose combinations with at least 6 measured timepoints after quality control were included. Out of the these all eight timepoints were measured in most time series, while only six timepoints were measured in two time series, and only seven timepoints in seven time series. “

“Fig 3: Enrichment of known events in DILI before adverse histopathology based on gene sets as well as individual gene members.” When you state gene sets, which comparisons did you perform? It is not clear from the writing.

In response to this comment we have added more information in the caption and also linked to Methods beforehand in main text now.

“Fig 4: Enrichment of known events in DILI before adverse histopathology based on gene sets as well as individual gene members. The enrichment of first activation before or at adverse histopathology is shown for gene sets mapping to known key events in DILI, for which first activation was defined as first timepoint of differential GSVA-derived gene set activity. Furthermore, also enrichment of individual genes within these genesets is shown and was derived based on the first timepoints of differential expression.”

## Reviewer #3

Reviewer #3: This study has several strengths, including the time-resolved transcriptomics demonstations. Importantly, the authors should be commended for their comprehensive efforts. The manuscript is clear and easy to read.

The authors thank the referee for their generally positive feedback.

A discussion of the results in relation to the more general issue of the TFs and Drug-Induced Liver Injury is lacking.

While this comment is somewhat unspecific and hence difficult to address we have in response extended our discussions as follows.

We have added some information on why DILI is relevant (page 5, l.115 ff):

“This is for example true in the context of DILI [14–18], which is a major cause for attrition in drug development and accounts for around half of the cases of acute liver failure in the US and European countries [19,20].”

We also provide more general details on why we focus on TFs (page 16, l.426 ff):

“To gain insight into signalling and expression regulation preceding adverse histopathology, we next analysed transcription factors (TFs), which are involved in early perturbation response preceding downstream gene expression changes and also are likely to show strong signal in transcriptomics data given their direct link to gene expression.”

Additionally, we extended the discussion on the roles of identified TFs in DILI, (page 19, l.516 ff):

“EIF2a is part of the same branch of Unfolded Protein Response (UPR) as Atf4 and causes its preferential translation which, among others, mediates autophagy and proapoptotic response [42,51] and is a known predictor of DILI [52]. Furthermore, increased folding of actin by Chaperonin containing tailless complex polypeptide 1 (CCT) or tailless complex polypeptide 1 ring complex (TRiC) is found frequently and with large effect size. It has been previously linked to proteostasis and autophagy [53,54], but a role in DILI specifically is not yet known. ”

## Reviewer #4

Reviewer #4: First, I’d like to thank Dr. Gallo, associate editor of PLOS Computational Biology, the opportunity to provide a peer-review for the work “Deriving time-concordant event cascades from gene expression data: A case study for Drug-Induced Liver Injury (DILI)” by Anika Liu and collaborators.

“Adverse outcome pathway, or AOP,” is a conceptual framework in toxicology that attempts to link a molecular initiating event with a defined adverse outcome. Liu and collaborators propose to identify novel potentially causal pairs of AOP-framework related key events (KE) and adverse outcomes (AO) by means of analysing temporal associations between these elements in an automated, data-driven way, with the aim of aiding in identifying novel AOPs in the future.

The authors propose to achieve this goal by using several metrics obtained from a time series dataset of liver gene expression deposited in the TG-GATEs database. These data, together with liver histopathologies, were obtained from the livers of rats that had been treated with a battery of xenobiotics. After normalising the microarray gene expression data, the authors used the first significantly differentially expressed gene as potential KE and capitalised on a previous exploitation of the same database that rendered scores of the histopathologies (as the AOs) to construct a model that captures the temporal dimension of the Bradford Hill criteria for causality [ref. 7 in the author’s manuscript] to prioritise associations with some support of causality. Being a pilar motivation of this work to capture causality in the associations detected, the authors limited their usable data to the perturbations, histopathology findings, and pathways that were relevant for the DILI pathology. This necessarily affected the capacity to generate pathway hypotheses that were purely data-driven, which is a central motivation of this work. However, despite the shortcomings that the authors nicely describe in a “Limitations of this study” section, they did a great job exploiting gene expression data to derive multiple metrics (significant enrichment, logFC and frequency/TPR) that combined maximise the ability to prioritise first event-outcome pairs from the database. This work is thus a valuable contribution to the global effort currently being undertaken to maximise the ability to exploit datasets deposited in countless databases, possibly serving not only as inspiration for novel ways of extracting important information from data, but also as a primer that can be further developed. Although I am not an expert on the toxicology domain, I believe that, given the linearity of the definition of AOP (Ankley et al., 2010 [ref. 5]; Leist et al, 2017 [ref. 4]), and the fact that it does not require full knowledge of the mechanism or pathway, the proposed method in this study is appropriate for its ambition of helping to establish new AOP hypotheses.

The authors thank the referee for their generally positive comments regarding this work.

That said, I believe the readership would enjoy of a better flow if the descriptions of the datasets, assays and methods were more detailed. For example, although the authors make a substantial depiction of the extracted data on Figure 2C, which includes insight on the TG-GATEs assays study design, I still fail to find there or elsewhere in the manuscript important information: it does not include details like number of replicates performed per time-series, a description of how the serial or ‘’repeat-dose” treatment of animals was performed, what kinds of controls were used, how the rat livers were collected for histopathology and for gene expression measurement, strains of the animals, how much (or what fraction) of the dataset was excluded from the current analysis, etc… A supplementary figure detailing the TG-GATEs study design could be sufficiently effective in facilitating a better understanding of the methodology that was subsequently applied in this study.

We have now included Fig S1to outline the study design and provide additional information in the caption:
“S1 Fig. Open TG-GATEs study design. 6-week-old male Crl:CD Sprague-Dawley (SD) rats were treated with a range of compounds using daily repeat-dosing. For each compound, four doses were used including a vehicle control, and samples were taken at 8 timepoints. For each combination of compound, timepoint and dose, histopathology was annotated and gene expression measured for 3 replicates.”

Likewise, the authors should be more explicit in describing parameters and formulas, specifically of the hypergeometric distribution they used in their models. Additionally, they may want to describe in more detail some of the packages used. For example, on page 29 line 577, the authors refer the usage of the Limma package. The narrative could be more intuitive for the general audience if the authors add a brief description of methods applied.

We have extended the description to clarify what limma was used for and what was compared (p.32, l.884):

To evaluate which pathway or TF is dysregulated, we computed the differential activity in comparison to the vehicle control group, which was treated for the same amount of time and as part of the same experiment, using the moderated t-statistic in limma [79].

We also provide additional details on the hypergeometric test (p.33, l.921ff):

“We then computed the enrichment of PE before or at LE using the fisher.test function of the stats R package [83], first estimating the odds ratio using the conditional maximum likelihood estimate and subsequently testing the null hypothesis whether the odds ratio derived from a confusion matrix as described in Fig 1 is equal to or smaller than 1. “

The figures and tables in the main document and supplementary material are good and generally adequate, but the authors may want to add a Q-Q plot associated with figure 3 to show the calibration of their hypothesis test.

A Q-Q plot would be useful to see if the observed distribution of p-values follows expected one. However, this can only be performed if the expected p-value distribution can be estimated, e.g. assuming that the null hypothesis should only not hold true for a small subset of tests resulting in a largely uniform distribution of p-values. In our case, however, we believe that this assumption is inappropriate as we expect many pathways and TFs to be involved in such a complex organ-level phenotype as DILI, and so we have chosen not to add Q-Q plots to it.

The authors should also be more detailed and descriptive in some of the legends. I list below some cases that could be improved (e.g., adding a legend to File S2, and a better description of Table S2), but maybe the authors will find more opportunities of improvement at other instances.

We have improved the captions of Figures 3, 4, 6, 7 and 9, as well as S2 Table and S4 Figure. We added an extra sheet explaining the columns to File S2, and improved the legends for Fig 9, and 10. (Outlined in more detail below.)

The abundance of detail in presenting their findings of the case study overshadows the core contribution of a novel methodology to derive the time-concordant events in the first place. The manuscript would benefit greatly if those two aspects would be presented each with more focus. One way of achieving this could be to generalise the examples that the authors curated into schematic models that highlight the strengths of the model: where in the generalized examples is the model contributing that a non-data-driven, non-hypothesis-free method would not.

We thank the reviewer for this positive comment and have added a subfigure 1C to outline this.

Finally, I believe that the authors will have a more engaged audience, enjoying of a better reading flow and understanding of the reasoning intended after the authors make a new round of careful editing, either themselves or with the help of a professional scientific editor.

I share below more specific comments and suggestions, by section in the main document. Note that the comments are not ordered by importance, but by approximate order of appearance in the manuscript. Where only a subsection tittle is shown, it means there are not further comments for that section or figure.

We’d like to thank the reviewer for their positive feedback and for their extensive efforts to provide us with constructive comments to further improve the quality of this manuscript. The points above have been addressed as described below.

Abstract

1. Adverse Outcome Pathway is not generally known. I suggest the authors to link the expression “Adverse Outcome Pathway” with the term “toxicity” early in the abstract (optimally in first sentence), if they intend to help the reader immediately identify the domain of the work in the main abstract.

This has been added.

2. Page 2, line 33-34: When referring to “significance, frequency and log fold change (logFC)”, the authors may want to specify that “gene expression” is the object of the metrics they are referring to.

This has been added.

Introduction

3. Acronym inconsistencies:

Page 4, line 72: The AE acronym has not yet been introduced/defined in my version of the manuscript. The authors may want to define it here.

This has been added.

Page 4, line 76: The acronym for mode of action is MOA (as seen in Meek, 2014), therefore “mode of action” should include capitalisation of the three words, or no capitalisation at all. It could also be contained in brackets.

This has been added.

Page 4, line 87: Defining an acronym with another acronym may be too distracting for the reader. Maybe the authors would like to replace “KE relationships (KERs)” with “key event relationships (KERs)” for the sake of text fluidity/readability.

This has been added.

4. Definition inconsistencies:

Page 4, lines 77-80: The description of the criteria to evaluate plausibility may be confusing, in particular the explanations in parenthesis. It is not clear what “A” and “B” refer to. Later in the manuscript, “A” refers to a key event and “B” to an adverse outcome. This does not seem to be what the authors intend to convey here. For example, to describe “incidence concordance” the authors write “(The magnitude of event A is larger than that of event B)”. However, a quick check of the referenced work of Meek et al, 2014 shows incidence is explained with the question: “Is the occurrence of the end (adverse) effect less than that for the preceding key events?”. I fail to interpret the two explanations to mean the same.

We thank the reviewer for pointing out the unclear definitions. In the introduction we now refer the E_cause_ and E_consequence_ to avoid confusion with the later definitions only based on time concordance alone (which may not be causal as discussed in the limitations). Indeed, E_cause_ (previously A) can be a key event and E_consequence_ (previously B) can be an adverse outcome. We have stated these as possible examples, but introduced the additional definitions of E_cause_ and E_consequence_ because relations are not limited to particular types of events. We also see how “magnitude” may not be the best description. We’ve rephrased this as “E_cause_ affects a larger population and is hence more frequent than E_consequence_”.

It could be that I am completely missing the point of the authors, but for the sake of clarity and fluidity, the authors may want to put the general parameters/events described in the intro in context of the actual models and kind of data that is being presented throughout the manuscript (if needed be, with examples).

On identical note, in Figure 1 and across the manuscript, the authors may want to standardise the definitions of: “A”, “B”, “event”, “early event”, “later event”, “potential preceding event”, “potential later event”, “key event”, “anchoring event”, and “outcome”. It is not intuitive to use the word “event” for the “later event” and, if the authors believe it makes sense, they may want to distinguish “event” when it means “early event” from when it means “later event” by using e.g. the word “outcome” instead, across the manuscript, figures and legends, for the later (i.e., use “outcome” every time the word “event” is used in the sense of the “later event”). If, for some sensible reason this is not advisable, at least the word “event” should always be accompanied with the consistent (always the same) preceding word, e.g., “early” and “anchoring” (or “later”).

Thank you for this comment, please see our response to the related next question below.

5. Figure 1 (Page 6, line 114):

Panel 1A: I understand that “A”,”B”,”C” and ”D” in panel 1A are instances of “A” in the model (“early event” or “potential preceding event”), as defined in the panel 1B, while “B”, in panel 1B (the model), is the “potential later event”. In Panel 1A, “A”,”B”,”C” and ”D” could be replaced by e.g., “I”,”II”,”III”, and “IV”. This way, “A” should always represent “preceding event” and “B” “later event or outcome” in the figure and across the manuscript.

Panel 1B) As already stated, in case the authors also believe it makes sense, they may want improve readability and consistency by defining “B” as “potential later outcome”.

We thank the reviewer for pointing out the unclear definitions of A and B in Fig. 1. We have now changed the notation for when referring to the potential preceding event (now "PE”, previously “A”) and for the potential later event (now “LE”, previously “B”) throughout the manuscript. Now letters A-D are solely used as examples in Fig.1A. We have also simplified the event definitions, replacing “anchoring event” with “later event”, and “earlier event” with “preceding event”. We did not replace “later event” with “outcome” because in the TF analysis the later event is TF activity which would not be considered an “outcome” from a toxicological standpoint. However, we did suggest It as alternative definition “later event or outcome” for the sake of clarity (p.33, l.911).

Results and Discussion

Adverse histopathological findings and their temporal relation

6. Notation inaccuracy

Page 7, line 150: I believe the authors are referring to intervals, but the notation used is ambiguous. Using explicit intervals will help readership: “null” is within (0, 0.67] and “low” is (0.67, 1.34] ?

A clarifying sentence was added (p. 8, l. 209):

“These toxscores were used to define three levels for each histological finding: “null” (toxscore > 0), “low” (toxscore > 0.67) and “high” (toxscore >1.34). For example in case of a toxscore of 1, both “null” and “low” are considered to be present.”

We also included the thresholding visually in the newly added workflow figure Fig. 2

7. Typos

Page 7, line 138: The authors may want to use the plural of the word “hypothesis”, i.e., “hypotheses”

Page 8, line 163: The subject seems to be missing at the end of the excerpt: “labels were next used to define 61 time-series associated”.

Both were corrected.

Page 8, lines 165 and 166: If by “insult” the authors mean “adverse outcome”, it would be better to use the latter.

Replaced with “adverse phenotype”.

8. Figure 2 (Page 8, line 168)

Panel 2A: Colors are hard to distinguish: authors may want to increase label circles (in legend) and consider to change colors or, since there seems to be pairs of harder to distinguish colors, add a pattern to one within the pair.

We have changed the colours used to make them easier to distinguish, and also increased the circles in the legend.

Figure 2 legend: Page 9, line 172: see comment for Page 7, line 150

This is in fact correct (extended description in Methods now and illustrated in workflow figure).

Page 9, line 174: panel 2B does not show frequencies, but number of conditions.

We mean by frequency the number of conditions, but have now included both terms in the caption to make clear that these are used interchangeably.

Page 9, line 177: numbered reference missing after “Sutherland et al”.

This has been added.

Known pathways in DILI preceding adverse histopathology

9. Typos and omissions

Page 9, line 185: replace “(Fig 3).” With: “(Fig 3 and Table S3).”

Fig 3 was added to the preceding mention of Table S3, and this cross-reference was removed.

Page 10, line 208: perhaps authors want to replace “processes except Growth Arrest And (…)” with: “processes except genes involved in Growth Arrest and (…)”

“Growth Arrest And DNA Damage-Inducible Protein” is the name of the gene, but the gene symbol was moved forwards to avoid this confusion.

10. Figure 3 (Page 10, line 193)

- It would be useful to show how well the data is following the model: to that effect, the authors may want to add Q-Q plots to this figure.

A Q-Q plot would be useful to see if the observed distribution of p-values follows expected one. However, this can only be performed if the expected p-value distribution can be estimated, e.g. assuming that the null hypothesis should only not hold true for a small subset of tests resulting in a largely uniform distribution of p-values. In our case, however, we believe that this assumption is inappropriate as we expect many pathways and TFs to be involved in such a complex organ-level phenotype as DILI, and so we have chosen not to add Q-Q plots to it.

Figure 3 legend:

Page 10, line 197: The LXR acronym is not defined anywhere in the manuscript.

This has been added.

11. Figure 4 (Page 12, line 229)

- This plot attempts to illustrate the strength of pathway perturbation. For the first panel, the authors may want to display the distribution of the “all” datapoint. This could be achieved by superimposing violin instead of box plots for this panel in particular. (The same extra information could be displayed in other figures anytime that the data is not sparse, usually, when the “all” datapoints are presented - Figure 3, Figure 6A and 6C).

This was added except for Fig 6A because the plot was too crowded with the violin plot.

12. Figure 5 (Page 13, line 239) and related Figure S3 (Supplementary file)

- It could be interesting to see how the data vary with varying FDR (actual precision-recall curves).

As we show in Fig. 4, the known events are indeed not all among the most significant ones. While a precision-recall curve would be feasible, we believe that this would focus too much on significance while we characterize time concordance through multiple metrics, e.g. also true positive rate and specificity, and discuss how these relate to different biological roles.

- Note that figure 5 is repeated, at least on my copy of the document: Page 13, line 239, line 242 and page 14 line 254 show the same image.

The formatting error was corrected.

Figure S3, related with Figure 5 (Supplementary file)

- Although these plots expose a number of metrics, it is not intuitive what one can learn from them. The authors may want to expand a bit more.

We assume the referee refers to Fig S4, where it fits better, instead of Fig S3. Here we indeed show quite a lot of metrics and we have extended the caption to explain each subfigure now:

“S4 Fig. Background distribution of temporal association metrics across pathway and Transcription Factor (TF) events. The dependency between different metrics is shown. A) Frequency of events by median max. |logFC| before or at histopathology and enrichment p-value. B) Frequency of events by true positive rate (TPR) and positive predictive value (PPV) before or at adverse histopathology. C) Direct relation between TPR, PPV and enrichment p-value. D) Direct relation between TPR, PPV and frequency in background time-series.”

Known TFs in DILI preceding adverse histopathology

13. Figure 6 (Page 16, line 297)

Figure 6 legend:

- It is not clear what “all” refers to (all the TFs in the genome?).

This has been added: “As background distribution in grey, the statistics for all inferred TFs is shown.”

14. Add reference to a figure in text

Page 15, line 292: In the sentence “(…)as most frequent event indicating that the(…)”, the authors may want to add a reference to Figure 6B: “(…)as most frequent event (fig.6B) indicating that the(…)”

Added this and another reference to Fig 6 at a different location in the text.

Data-driven prioritisation of cellular events taking place before adverse histopathology

15. File S2 (Supplementary file)

Page 16, line 311: File S2 - Is a supplemental spreadsheet, first referred on Page 10, line 202 - it would be useful if the authors added a legend describing the columns names, e.g., in a new tab.

A separate tab has been added with descriptions of the column metrics as well as information on how these metrics were derived.

16. Typos, clarification and nomenclature consistency

Page 17, line 323: In addition to the acronym, “UPR” should be described.

This has been added.

Page 17, line 320-331: The authors may want to contextualize the analysis described in the 4 sentences starting in “The most frequent(…)” and ending in “(…)the most strongly down-regulated TFs.” relative to their inclusion as known or plausible events in a more specific way, in order to cater for the readership that is not expert in DILI.

We have extended the results section and clarified this part of the text (p.18, l. 503ff).

*“The most frequent genesets point to translation regulation via Eukaryotic translation initiation factor 2A (EIF2a) including the upstream response mediated by eIF-2-alpha kinase GCN2 and the downstream role in protein translation mediated through interactions with tRNA. EIF2a is part of the same branch of Unfolded Protein Response (UPR) as Atf4 and causes its preferential translation which, among others, mediates autophagy and proapoptotic response [42,51] and is a known predictor of DILI [52]. Furthermore, increased folding of actin by Chaperonin containing tailless complex polypeptide 1 (CCT) or tailless complex polypeptide 1 ring complex (TRiC) is found frequently and with large effect size. It has been previously linked to proteostasis and autophagy [53,54], but a role in DILI specifically is not yet known. As most strongly dysregulated events, metabolic pathways are found pointing to increased beta oxidation of fatty acids, as well as decreased cholesterol biosynthesis and tyrosine catabolism. Also the most strongly down-regulated TFs point towards lipid metabolism, i.e. the Sterol Regulatory Element Binding Transcription Factor 1, Srebf1, the Sterol Regulatory Element Binding Transcription Factor 2, Srebf2, as well as as well as Nr1h3 which controls Srebf1 expression. Overall, the derived time concordant events, which take place between the beginning of treatment and onset of adverse histopathology, hence include known and plausible events in liver injury which can be further characterized based on their frequency, significance and logFC.”*

Page 17, line 329: Gene names: please make sure you use this journal’s gene names convention and use it consistently across the manuscript.

We believe this targets the introduction of Srebf1 and Srebf2 and have clarified this now: “the Sterol Regulatory Element Binding Transcription Factor 1, Srebf1, and the Sterol Regulatory Element Binding Transcription Factor 2, Srebf2”

Confusion around gene names in this manuscript might stem from two facts, namely that we usually refer to rats (Only first letter in gene symbol is capitalized) but sometimes cite literature on findings in humans (All letters in gene symbol are capitalized). Additionally, when discussing inferred TF activity we consider these as proxy for proteins (Not italicized) while we refer to the genes when discussing expression (italicized). We’d like to point to previous literature as reference for this naming convention ([10.1007/s00335-010-9284-4](https://dx.doi.org/10.1007%2Fs00335-010-9284-4))

Figure 7

Identifying mechanistic hypotheses combining known TF functions and time concordance

17. TF activity metrics clarification

- The authors may want to explain how regulon activity is measured, or what is the output of the packages that extract this data (here or/and in the material and methods section, see my point 25.1).

Extended in methods (p. 32, l.873).

18. Figure 8 (Page 19, line 359)

Figure 8 B: It’s unclear what the units for transcription factor activity are or mean.

In response to this comment we have added a more descriptive legend and axis labels, as well as an extended caption as follows:

“Fig 8: Transcription Factor (TF) activity and expression before adverse histopathology. A) Significance of enrichment in adverse conditions for matched TF activity and expression-based events. Events only found on the expression or TF level are not included in the figure due to the absence of a statistical test for those. B) For significantly enriched TF activity-based events, the True Positive Rate (TPR) of observing TF activity before or at the time of adverse histopathology is shown, as well as the TPR for observing TF expression changes before TF activity in the time series where it precedes adverse histopathology.”

19. Figure 9 (Page 21, line 408)

19.1 - It’s hard to distinguish what is diamond from circles because the gene names prevent visibility; perhaps the shapes size could be increased, for example, or/and the names could be non-superimposing the shapes, or the color scheme changed.

19.2 - The shadings of grey are nearly indistinguishable for the important metrics of “Only at the same time” or “Ordered or at the same time”, authors may want to use a different color scheme

We have changed the color palettes for nodes and edges, to make them more distinguishable.

Time-concordant events reflecting disease progression

20. Figure 10 (Page 23, line 444)

Figure 10 legend:

Page 23, line 448 - typo in word “correlation”

Corrected.

Limitations of this study

Conclusions

21. Transforming specific pathway examples into a generalised model schematics depicting the strengths (and possibly shortcomings) of the method:

In addition to detailing the pathways themselves, it would be more useful to the readership interested in the methodology to use generalised terms that would explain the capabilities of this model. One way the authors could achieved this is by drawing a schematic illustrating how the author’s method was able to confirm an example of known events in DILI, and contrasting it with other generalised example(s) where the model was able to fill in the gaps in knowledge, at different levels.

We have added a subfigure C to Figure 1 to illustrate how time concordance can generally be used, namely to add information on particular links between events or to prioritize mechanistically linked events.

22. Page 27, line 524: Link to Shiny app is missing at this location

This has been added.

Methods

Open TG-GATES data processing

Definition of adverse histopathology

23. Expand on methodology details

Page 28, line 563: More details should be given. Please be explicit about the parameters and equation used in the hypergeometric test. Also, please provide intuition for the choice of hypothesis test (i.e. why the hypergeometric distribution).

We have added more details on how the confusion matrix was generated and how the test was performed (p. 31, l.853):

“All labels which were identified with these criteria are significantly enriched among time-series labelled as adverse by Sutherland et al. in comparison to those that were considered non-adverse using a one-sided Fisher’s Exact test (p-value > 0.0001), performed using the fisher.test function of the stats R package. “

We have not included additional information on why this exact test was chosen, as we believe this shifts the focus away from the main analysis (The results of the test are not used for filtering but only to support the implemented criteria). However, it is suitable for the small sample sizes at hand and widely used in related applications, e.g. in clinical context ([10.5395/rde.2017.42.2.152](https://dx.doi.org/10.5395%2Frde.2017.42.2.152)) or for gene set analysis ([10.3389/fgene.2020.00654](https://doi.org/10.3389/fgene.2020.00654)).

Page 28, line 559: The authors may want better define DILIst, DILIrank and explain table S2 further.

We have added a bit more explanation on these databases and also included the references now (p. 32, l.367):

“While not all compounds in the TG-GATEs database are drugs and some mechanisms of toxicity may not translate to humans, out of the 38 compounds represented in adverse time-series, 22 have additionally been classified as hepatotoxic in DILIst [28] and 18 in DILIrank (vMost-DILI-Concern or vLess-DILI-Concern) [27] (S2 Table). This overlap with compound-level DILI annotations by the FDA shows that the compounds in this study partially represent known mechanisms of DILI in humans, while also highlighting the fact that a clear classification is not possible.“

We also extended the caption of S2 Table:

“S2 Table. Compounds classified as adverse based on histopathology and concordance with previous annotations. For the annotations by Sutherland et al. [15], who classified each compound at each measured dose as adverse or non-adverse at day 4 and day 29, the adverse doses for each compound are listed. Furthermore, the binary classification as adverse (1) and non-adverse (0) from DILIst [84] are included as well as the vDILIConcern and Severity Class classifications from DILIRank [85] which describe evidence for liver side effects observed in humans derived from post-marketing data. “

Page 27, line 539: Typo: Replace “To characterize the extend…” with “To characterize the extent…”

Done.

Page 27, line 542-3: And replace: ”(…)and then summarised across all biological replicates by mean as an aggregate(…)” with: “(…)and then averaged across all biological replicates as an aggregate(…)”

Changed.

Page 27, line 550: see comment 6. (Page 7, line 150)

Addressed above.

Pathway and TF activity inference

25. Expand on methodology details

25.1 - Page 28, line 567-8: The authors may want to give a more granular description of the TF activity output by the package(s) mentioned. How does TF activity data look like? It could also be useful to include a short explanation of the choices made.

We have extended the description as follows (p. 32, l.374):

“The activity of pathways and TFs across all doses and timepoints of a treatment including vehicle controls was derived based on the expression of its gene sets members using GSVA [26], which computes a gene set enrichment by sample matrix from the gene expression by sample matrix. This was performed using a Gaussian kernel requiring at least 5 genes per gene set, and overall provides the basis for the subsequent pathway- and TF-centric steps”

25.2 - Page 28, line 575-7: The authors may also want to exactly describe which methods were used in addition to the packages that assisted on the implementation of those methods.

We have extended the description to clarify which test was used and what was compared (p.32, l.884):

To evaluate which pathway or TF is dysregulated, we computed the differential activity in comparison to the vehicle control group, which was treated for the same amount of time and as part of the same experiment, using the moderated t-statistic in limma [79].

25.3 - A thorough description of the TC-GATEs dataset must also be shared in order to effectively understand the analysis. As suggested earlier, this could include a figure.

We now included information on the TG-GATEs dataset in the new Fig. S1. and its caption:
“Open TG-GATEs study design. 6-week-old male Crl:CD Sprague-Dawley (SD) rats were treated with a range of compounds using daily repeat-dosing. For each compound, four doses were used including a vehicle control, and samples were taken at 8 timepoints. For each combination of compound, timepoint and dose, histopathology was annotated and gene expression measured for 3 replicates.”

25.4 - In addition to a schematic figure describing the assays, it would be most useful to include more schematics of the author’s pipeline.

This has now been added as Fig 2.

Temporal concordance of events

26. Page 30, line 598: The authors may want to share the parameters and equation used in the hypergeometric test.

We now provide additional details on how this was implemented (p.33, l.921ff):

“We then computed the enrichment of PE before or at LE using the fisher.test function of the stats R package [83], first estimating the odds ratio using the conditional maximum likelihood estimate and subsequently testing the null hypothesis whether the odds ratio derived from a confusion matrix as described in Fig 1 is equal to or smaller than 1. “

## General comment

During the revisions, we noticed an error in the gene-level time concordance statistics as some time-series were wrongfully included as background time-series. As a consequence, this affects the statistics for gene-level events shown in Fig. 4 and 6 (although the overall trends remain), as well as the inference of induced TFs shown in Fig. 9. While Irf9 and Pbx2 not being considered induced anymore, while Cebpd, Klf4, Meis2 and Nfkb1 are additionally considered induced. Out of these Meis2 and Nfkb1 also show known interactions which are now also shown in Fig. 10. As the pathway- and TF-level statistics were not affected by this error, the rest of the analysis remains unchanged.

In the text, the discussion of the top 10 most significant genes has been corrected (p.12, l.361 ff), as well as the discussion of induced TFs (pages 21-23) so that all display items as well as the main text are correct and in sync.
